# Analyzing health inequality among adolescents in Chile: Physical activity, socioeconomics, and play environments across genders

**DOI:** 10.1016/j.puhip.2025.100666

**Published:** 2025-10-08

**Authors:** Josivaldo de Souza-Lima, Maribel Parra-Saldías, Sandra Mahecha Matsudo, Gerson Ferrari, Andrés Godoy-Cumillaf, Claudio Farias-Valenzuela, Pedro Valdivia-Moral

**Affiliations:** a Facultad de Educación, Universidad de Granada, Spain; b Facultad de Educación y Ciencias Sociales. Instituto del Deporte y Bienestar, Universidad Andres Bello, Las Condes, Santiago de Chile, Chile; c Departamento de Educación Física, Deporte y Recreación, Universidad de Atacama, Chile; d Especialidad en Medicina del Deporte y Actividad Física, Facultad de Medicina y Ciencias de la Salud, Universidad Mayor, Santiago, Chile; e Centro de Investigación en Medicina, Deporte, Ejercicio y Salud. Clínica MEDS, Santiago, Chile; f Universidad de Santiago de Chile (USACH), Escuela de Ciencias de la Actividad Física, el Deporte y la Salud, Santiago, Chile; g Grupo de Investigación en Educación Física, Salud y Calidad de Vida (EFISAL), Facultad de Educación, Universidad Autónoma de Chile, Santiago, Chile; h Escuela de Ciencias de la Actividad Física, Universidad de Las Américas, Santiago, 7500975, Chile

**Keywords:** Youth well-being, Gender-specific analysis, Recreational safety, Physical activity disparities, Socioeconomic determinants, Adolescent health equity

## Abstract

**Objectives:**

This study examines the association between socioeconomic factors, gender, physical activity, and health satisfaction among Chilean adolescents. It aims to identify key disparities and their implications for public health policies.

**Study design:**

Cross-sectional study based on the third wave (2016–2019) of the international Children's Worlds survey.

**Methods:**

A total of 911 adolescents aged 12–13 years from Santiago, Chile, participated. Physical activity levels, socioeconomic indicators, and health satisfaction were assessed. Statistical analyses included t-tests, chi-square tests, ANOVA, and multiple linear regression models stratified by gender.

**Results:**

Boys reported higher levels of electronic gaming (64.6 % vs. 35.4 %, p < 0.001), while girls engaged more in outdoor play at lower frequencies (52.8 % vs. 47.2 %, p = 0.045). Perceived safety was a stronger predictor of health satisfaction for girls (β = 0.252, p < 0.001) than for boys (β = 0.251, p < 0.001).

**Conclusions:**

Socioeconomic and environmental factors significantly influence adolescent health satisfaction, with gender-specific differences. Policies should focus on improving access to recreational spaces and addressing economic barriers, particularly for girls.

## 1 What this study adds

• This study highlights gender-specific disparities in physical activity and health satisfaction among Chilean adolescents, showing that girls are more affected by perceived safety and access to recreational spaces.
• It demonstrates the stronger role of socioeconomic factors, such as access to money for school trips, in predicting health satisfaction for boys compared to girls.
• The research fills a gap in literature from middle-income countries by using secondary data from the Children's Worlds survey to link environmental factors with adolescent well-being.

## 2 Implications for policy and practice

• Public health policies in Chile should prioritize improving safety and accessibility of recreational spaces to boost physical activity, especially for girls from lower socioeconomic backgrounds.
• Interventions targeting socioeconomic barriers, like subsidies for school trips and technology access, could enhance health equity among adolescents.
• Gender-tailored programs promoting outdoor play for girls and structured exercise for boys may improve overall health satisfaction and reduce inequalities.

## 3 Introduction

Adolescence represents a critical stage in human development, where behavioral patterns and habits acquired have a significant impact on health and well-being throughout life [1,2]. Participation in physical activities during this period is widely recognized as a cornerstone for promoting comprehensive health [3,4]. Beyond the obvious benefits for physical fitness and motor skills, regular physical activity is also associated with important psychosocial advantages, such as improved academic performance, enhanced self-esteem, and reduced depressive symptoms [4,5] These positive effects extend not only to preventing diseases associated with sedentary lifestyles, such as obesity, but also to fostering emotional well-being, emotional regulation, and effective social interaction [6,7] (see Table 1, Table 2, Table 3).

**Table 1:** Distribution of sociodemographic characteristics, family resources, and physical activity patterns by gender in the study sample.

Variables	Boy	Girl	Total	p-value
Number of participants (%)	488 (53.6)	423 (46.4)	911 (99.0)	0.034
**Age**				
12 years old	303 (53.3)	265 (46.7)	568 (100.0)	0.862
13 years old	185 (53.9)	158 (46.1)	343 (100.0)	
**Family has a car**				
No	117 (57.6)	86 (42.4)	203 (100.0)	0.157
Yes	363 (52.0)	335 (48.0)	698 (100.0)	
**Family has telephone**				
No	16 (55.2)	13 (44.8)	29 (100.0)	0.839
Yes	466 (53.3)	409 (46.7)	875 (100.0)	
**Have access internet**				
No	33 (58.9)	23 (41.1)	56 (100.0)	0.384
Yes	450 (52.9)	400 (47.1)	850 (100.0)	
**Have pocket money**				
No	104 (56.5)	80 (43.5)	184 (100.0)	0.352
Yes	373 (52.7)	335 (47.3)	708 (100.0)	
**Family has Computer**				
No	27 (60.0)	18 (40.0)	45 (100.0)	0.365
Yes	456 (53.1)	403 (46.9)	859 (100.0)	
**Have money School Trips**				
No	71 (57.3)	53 (42.7)	124 (100.0)	0.362
Yes	407 (52.9)	363 (47.1)	894 (100.0)	
**Area safe walk**				
I do not agree	24 (54.5)	20 (45.5)	44 (100.0)	0.019
Agree a little bit	42 (39.3)	65 (60.7)	107 (100.0)	
Agree somewhat	73 (52.5)	66 (47.5)	139 (100.0)	
Agree a lot	111 (59.4)	76 (40.6)	187 (100.0)	
Totally agree	225 (55.1)	183 (44.9)	408 (100.0)	
**Area places to play**				
I do not agree	45 (58.4)	32 (41.6)	77 (100.0)	0.316
Agree a little bit	33 (47.8)	36 (52.2)	69 (100.0)	
Agree somewhat	59 (49.2)	61 (50.8)	120 (100.0)	
Agree a lot	78 (60.0)	52 (40.0)	130 (100.0)	
Totally agree	263 (53.5)	229 (46.5)	492 (100.0)	
**Frequency play outside**				
≤2 time/week (%)	125 (47.2)	140 (52.8)	265 (100.0)	0.045
2-4 times/week (%)	191 (55.7)	152 (44.3)	343 (100.0)	
≥5 times/week (%)	168 (56.8)	128 (43.2)	296 (100.0)	
**Frequence Sports Exercise**				
≤2 time/week (%)	76 (43.7)	98 (56.3)	174 (100.0)	<0.001
2-4 times/week (%)	164 (48.4)	175 (51.6)	339 (100.0)	
≥5 times/week (%)	245 (62.0)	150 (38.0)	395 (100.0)	
**Frequency play electronics games**				
≤2 time/week (%)	36 (21.7)	130 (78.3)	166 (100.0)	<0.001
2-4 times/week (%)	117 (51.1)	112 (48.9)	229 (100.0)	
≥5 times/week (%)	331 (64.6)	181 (35.4)	512 (100.0)	

**Satisfied Health**	482 (8.99 ± 1.73)	420 (8.62 ± 2.18)	902 (100.0)	0.005

**Table 2:** Linear regression of factors associated with health satisfaction in adolescents.

Predictor	Estimate	SE	t	p-value
Constant	9.076	16.516	5.49	<0.001
Age	−0.215	0.1278	−1.68	0.093
Gender	−0.275	0.1257	−2.19	0.029
Computer at Home	0.361	0.2966	1.22	0.224
Money for School Trips	0.715	0.1830	3.91	<0.001
Safe Areas to Walk	0.252	0.0485	5.20	<0.001
Frequency Play Outside	0.309	0.0875	3.54	<0.001
Frequence Sports Exercise	0.214	0.0912	2.34	0.019

*Footnote:* p-values below 0.05 indicate a statistically significant relationship between the predictor and health satisfaction. Positive coefficients reflect an increase in health satisfaction with the predictor, while negative coefficients indicate a decrease. Interpretations should be made within the context of the study design and do not imply direct causality.

**Table 3:** Comparative analysis of health satisfaction determinants by gender.

Predictor	Coefficient (Boy)	p-value (Boy)	Coefficient (Girl)	p-value (Girl)
Constant	72.543	<0.001	9.076	<0.001
Age	−0.0896	0.546	−0.215	0.093
Computer at Home	−0.1668	0.579	0.361	0.224
Money for School Trips	10.684	<0.001	0.715	<0.001
Area places to play	0.2511	<0.001	0.252	<0.001
Frequency Play Outside	0.1721	0.114	0.309	<0.001
Frequency Sports Exercise	0.4150	<0.001	0.214	0.019

*Footnote:* The coefficients reflect the strength and direction of the relationship between each predictor and health satisfaction for male and female groups, respectively. A positive coefficient indicates a direct relationship, where increases in the predictor are associated with increases in health satisfaction, whereas a negative coefficient indicates an inverse relationship. The p-values denote the statistical significance of each coefficient, with values below 0.05 generally considered significant. The constant represents the estimated health satisfaction when all predictor variables are at zero.

In this context, specific behaviors, such as participation in team sports, regular exercise, and unstructured outdoor play, have been identified as particularly beneficial [8]. These activities promote key social skills, such as teamwork and social integration, while fostering emotional self-regulation and reinforcing a sense of personal achievement [[9], [10], [11], [12]]. Moreover, these benefits are amplified when children and adolescents have access to environments that encourage physical activity, such as parks, plazas, and other recreational infrastructures [13,14]. Research on the use of these built environments has shown that they are essential for promoting higher levels of physical activity, which in turn improve health outcomes and perceptions of well-being in this population [[15], [16], [17]].

Despite these benefits, disparities in access to and use of recreational spaces remain a significant challenge, particularly in regions characterized by substantial socioeconomic inequalities [[18], [19], [20]]. Differences in the availability and safety of these spaces can directly influence participation in physical activities, as well as children's perception of safety and health satisfaction. These inequalities often reflect and amplify underlying economic disparities, creating a cycle of exclusion that disproportionately affects children from lower-income families [13,19,21,22].

These barriers not only restrict opportunities for recreational activities but also negatively impact perceptions of health, a key indicator of subjective well-being and a predictor of future quality of life [[22], [23], [24]].

This phenomenon is particularly evident in countries like Chile, where pronounced socioeconomic inequalities significantly affect the equitable distribution of recreational resources. This provides an ideal context to explore how these dynamics influence health perceptions among Chilean adolescents [25,26]. This limits the generalization of the findings to contexts like Chile, where socioeconomic and gender disparities play a prominent role in shaping health behaviors [27].

In Chile, a country with deep socioeconomic disparities, differences in access to recreational resources and participation in physical activities reflect and amplify these structural inequities [28,29]. Children and adolescents from lower socioeconomic strata face greater limitations in accessing safe and adequate spaces for play and physical activity. These inequalities have significant implications for health perception, a key indicator of general well-being during childhood and adolescence [27]. Furthermore, these disparities are influenced by gender-related factors, as the behaviors and opportunities of boys and girls regarding play, sports, and physical activity are often shaped by cultural and social norms that perpetuate traditional gender roles [30,31].

Furthermore, these disparities are influenced by gender-related factors, as the behaviors and opportunities of boys and girls regarding play, sports, and physical activity are often shaped by cultural and social norms that perpetuate traditional gender roles [32]. These differences not only limit girls’ opportunities to develop physical and social skills but also affect their perception of well-being and health [33]. However, the literature on gender disparities in access to recreational resources in Latin America remains scarce, highlighting the need for further research to address this issue.

The present study aims to evaluate the associations between socioeconomic factors, gender, physical activity, and health satisfaction among Chilean adolescents. This work addresses a critical gap in the literature, as most previous studies on gender and physical activity have been conducted in high-income countries, limiting their applicability to contexts with structural inequalities characteristic of middle-income countries like Chile. By focusing on these dynamics, this research offers a unique perspective on the barriers and opportunities faced by adolescents in diverse socioeconomic contexts, contributing to a more global and equitable understanding of the impact of physical activity on health. The findings of this study have the potential to inform targeted interventions and public policies that promote health equity for adolescents in settings with significant socioeconomic inequalities [15].

## 4 Methods

This study utilizes secondary data from the Children's Worlds survey; thus, sample size was not calculated de novo but based on the original survey's stratified sampling to ensure representation across socioeconomic contexts in Santiago, Chile. The sample of 911 adolescents was selected to include at least 20 per age group, with stratification for diversity [[34], [35], [36], [37]]. The cross-sectional design employed here facilitates the analysis of key associations between socioeconomic variables, gender, physical activity patterns, and health perception among Chilean adolescents. This approach is particularly suited to capturing the interrelations between these dimensions within a defined temporal context, providing a representative snapshot of the socioeconomic and health conditions in this population.

## 5 Study population

The study population comprised a sample of 911 adolescents aged 12 and 13 years from the Metropolitan Region of Santiago, Chile. Data were collected during the project's third wave (2016–2019) and stratified to ensure adequate representation of diverse socioeconomic contexts. Inclusion criteria required participants to complete at least 90 % of the questionnaire, with a minimum of 20 adolescents per age group. Adolescents with incomplete data related to dependent variables were excluded. This age group was chosen due to its importance in studying a key stage of physical and psychosocial development when physical activity and health patterns begin to solidify.

This study complied with international ethical guidelines for research involving human subjects, with approval from institutional ethics committees in each participating country [[38], [39], [40], [41], [42], [43]]. In Chile, it adhered to national research ethics standards and the *Comité Asesor de Bioética de Fondecyt-CONICYT* principles, in line with the Declaration of Helsinki [44]. Informed consent from legal guardians and assent from children were obtained, with strict measures ensuring data confidentiality. The study followed CIOMS guidelines for research with minors, prioritizing participant safety and minimizing potential risks [45].

## 6 Variables and measures

### 6.1 Age

Age data were collected through a multiple-choice question where participants selected their age group. Only adolescents aged 12 and 13 years were included, enabling a focused analysis of a well-represented cohort.

### 6.2 Gender

Gender was assessed through a binary question with the options "A girl" and "A boy." This categorization enabled differentiated analyses between boys and girls, identifying disparities related to physical activity patterns and health satisfaction.

### 6.3 Socioeconomic status

Socioeconomic status was evaluated using indicators such as internet access, availability of money for school trips, and ownership of assets like a car, phone, or computer. These variables, collected via binary "Yes" or "No" responses, provide a comprehensive view of household economic status in the Chilean context.

### 6.4 Environment and safety

To evaluate perceptions of the environment, participants responded to statements such as "I feel safe walking in my area" and "There are enough places to play and have fun in my area," using a Likert scale ranging from "Strongly disagree" to "Strongly agree."

### 6.5 Physical activity and leisure patterns

The frequency of daily activities outside school hours, such as "Playing sports or exercising," "Playing or spending time outdoors," and "Playing video games," was assessed through questions with response options ranging from "Never" to "Every day." For this study, responses were categorized into three groups: "≤2 times/week," "2–4 times/week," and "≥5 times/week."

### 6.6 Health satisfaction

Health perception was measured using an 11-point Likert scale, where 0 indicated "Not satisfied at all" and 10 ″Completely satisfied." This indicator captures the subjective evaluation of adolescents' physical and mental well-being, offering a comprehensive approach to understanding their quality of life.

### 6.7 Statistical analysis

Statistical analyses were conducted using Jamovi (version 2.3). Descriptive analyses summarized the sample through absolute frequencies, percentages, and means ± standard deviations. Independent t-tests, Chi-square tests, and ANOVA were applied to assess differences in health satisfaction, resource access, and physical activity frequency. Multiple linear regression identified predictors of health satisfaction, considering gender, age, socioeconomic factors, and activity-related behaviors. Gender-stratified regressions further explored differences in determinants between boys and girls. Normality and variance homogeneity were verified using the Shapiro-Wilk and Levene's tests, while multiple imputations handled missing data.

## 7 Results

### 7.1 Sample characteristics

The sample consisted of 911 adolescents, 53.6 % of whom were boys and 46.4 % girls. The mean age was 12.4 years (SD = 0.48). Descriptive results revealed significant differences between boys and girls in access to resources and physical activity behavior. For instance, boys reported a higher frequency of electronic game use (≥5 times/week: 64.6 % vs. 35.4 %, p < 0.001), whereas girls showed slightly higher participation in outdoor play at lower frequencies (≤2 times/week: 52.8 % vs. 47.2 %, p = 0.045).

The linear regression analyses revealed that health satisfaction was significantly influenced by gender, physical activity frequency, and access to socioeconomic resources. This regression coefficient indicates that, holding other variables constant, girls reported 0.275 units lower health satisfaction on an 11-point scale compared to boys. However, exercise frequency emerged as a significant predictor for both boys and girls (*p* < 0.001 in boys, *p* = 0.019 in girls).

Access to money for school trips and the perception of safe areas to play were positive predictors of health satisfaction for both genders, though the effect was stronger in boys (Coefficient: 10.684, *p* < 0.001 in boys vs. 0.715, *p* < 0.001 in girls). Additionally, the frequency of outdoor play showed a stronger association in girls (Coefficient: 0.309, *p* < 0.001) compared to boys (Coefficient: 0.1721, *p* = 0.114).

When comparing the results of gender-stratified models, frequent exercise emerged as a stronger determinant for boys, whereas outside play and access to resources such as computers and safe areas had a relatively greater impact on girls. The chi-square test (p < 0.001) indicates a significant association between gender and electronic gaming frequency, with boys more likely to engage frequently.

## 8 Discussion

This study provides a comprehensive understanding of how sociodemographic, environmental, and behavioral factors influence health satisfaction among Chilean adolescents. The results highlight significant gender differences, with girls showing a greater reliance on perceptions of safety and access to recreational areas, while boys exhibit a stronger impact from structured physical activities such as regular exercise. These findings underscore the complex interplay between gender, available resources, and physical activity patterns in shaping subjective well-being.

The findings of this study align with prior research demonstrating that sociodemographic, environmental, and behavioral factors shape adolescent well-being. Reiner et al. (2013) reported that physically active adolescents are 20 %–30 % less likely to experience anxiety and depression, highlighting the mental health benefits of physical activity beyond its physical advantages [46]. Janssen and LeBlanc (2010) further noted that engaging in at least 60 min of moderate-to-vigorous physical activity daily is linked to greater perceived well-being, energy, and optimism, contributing to higher life satisfaction and academic performance [47]. Similarly, Eime et al. (2013) emphasized that organized sports enhance both physical health and psychosocial skills like teamwork and emotional regulation, aligning with this study's findings on the positive impact of exercise, particularly in boys (Coefficient: 0.415, p < 0.001) [48].

Music Milanovic et al. (2021) reported that girls from disadvantaged backgrounds face 65 % more barriers to accessing safe recreational areas compared to 45 % for boys. Additionally, boys with parents of lower educational attainment are 2.24 times more likely to engage in less than 2 h of organized sports per week. These findings underscore the role of socioeconomic disparities in shaping adolescent physical activity. Economic limitations were also linked to a higher likelihood of excessive screen time, affecting 38.4 % of boys with lower perceptions of family wealth [49]. The beta coefficient (β) of 0.252 for girls means that a one-unit increase in perceived safety is associated with a 0.252-unit increase in health satisfaction, controlling for other factors.

In line with this, the study by Oreskovic et al. (2015) found that adolescents who perceive their environment as safe are 30 % more likely to engage in outdoor recreational activities, highlighting the critical role of perceived safety as a determining factor in youth physical activity [50]. This slight difference suggests that girls rely more heavily on the perception of safety in their environment to engage in outdoor activities, reflecting gender-specific cultural and social barriers that limit their access to recreational spaces.

Outside play frequency had a stronger impact on girls (Coefficient: 0.309, *p* < 0.001) than on boys (Coefficient: 0.172, *p* = 0.114), aligning with previous studies highlighting the benefits of unstructured recreational activities for girls. Werneck et al. (2022) noted that girls have less access to public physical activity facilities, and the presence of such facilities increases the likelihood of recreational participation among low-income individuals by 207 % (OR: 3.07; 95 % CI: 2.35–4.01) [51]. This finding emphasizes that the perception of safety and the availability of public spaces are crucial factors for promoting physical activity among girls, who face greater cultural and social barriers.

Tandon et al. (2021) found that adolescents from high-income families are 3.08 times more likely to meet daily physical activity recommendations and that access to technology increases perceived well-being by 15 % when used for educational or recreational purposes. However, this study found no significant relationship between computer access and health satisfaction (coefficients: 0.361 in girls, −0.1668 in boys; p > 0.05) [52]. This discrepancy may reflect differences in technology use, where in Chile, computer access is more linked to academic responsibilities or excessive social media use rather than activities that promote well-being.

Finally, the findings on the role of access to money for school trips as a significant predictor for both genders (Coefficient: 10.684, *p* < 0.001 in boys; Coefficient: 0.715, *p* < 0.001 in girls) are consistent with studies in Latin America. Gutiérrez-González et al. (2023) reported that the prevalence of childhood obesity in low-income households was more than double that of high-income households (26.8 % in boys and 20.4 % in girls compared to 12.1 % and 8.7 %, respectively). Additionally, boys from disadvantaged families were 22 % less likely to participate in extracurricular activities due to economic constraints [53]. These figures emphasize how the lack of financial resources not only limits access to recreational opportunities but also exacerbates disparities in child health and well-being, particularly in economically vulnerable contexts.

## 9 Strengths and limitations

This study's strengths include its use of a large, stratified sample from the Children's Worlds survey, providing robust insights into Chilean adolescents. Limitations include the cross-sectional design, which prevents causal inferences, and reliance on self-reported data, which may introduce bias. Additionally, variables such as social support or family dynamics, which could significantly influence the results, were not included. Lastly, the findings may not be generalizable to other regions of Chile or countries with different cultural and economic contexts.

## 10 Future directions

Future research should consider a longitudinal design to assess how changes in socioeconomic and recreational factors affect health satisfaction over time. It would also be valuable to explore how gender-specific interventions could optimize participation in recreational activities and the perception of well-being. Incorporating additional variables, such as family relationships and social support, would provide a more comprehensive understanding of the factors that determine health satisfaction in this population.

## Ethical approval

This study is based on a secondary analysis of anonymized, publicly available data from the Children's Worlds study (International Survey of Children's Well-Being, ISCWeB), specifically the Chilean sample from the third wave (2016–2019). No new human subjects were recruited, no primary data were collected, and no identifiable personal information was used or processed in our analysis. As such, ethical approval from an Institutional Review Board (IRB) or Ethics Committee was not required for this research.

This is in accordance with Chilean national legislation, particularly Law 20.120 (2006) on scientific research involving human beings, which regulates direct interventions, biological samples, or personal data processing but does not mandate new ethical approval for secondary analyses of de-identified, aggregated datasets from previously approved studies. The original ISCWeB study obtained country-specific ethical approvals, including parental consent and child assent, aligned with the Declaration of Helsinki (as detailed in the ISCWeB Comparative Report 2020, pages 93–94). For the Chilean component, ethical procedures were managed through the Scientific Ethics Committee (Comité Ético Científico) at Universidad del Desarrollo. For further information on sampling and ethical procedures, national reports are available on the ISCWeB website: https://isciweb.org.

## Conflict of interest statement

We, the authors of the manuscript entitled "*Analyzing Health Inequality Among Adolescents in Chile: Physical Activity, Socioeconomics, and Play Environments Across Genders*", submitted to Public Health in Practice, declare that we have no financial, professional, or personal relationships that could inappropriately influence (or be perceived to influence) the work reported in this manuscript.

## Funding disclosure

This research was conducted without any external funding or financial support from organizations that could present a conflict of interest. If applicable, funding sources related to this study have been duly acknowledged in the manuscript.

## Author contributions and independence

All authors contributed significantly to the conception, design, execution, and writing of this manuscript. No external parties influenced the study's design, data interpretation, or conclusions.

## Ethical considerations

This study complies with ethical guidelines, and no conflicts of interest related to ethical misconduct or research integrity are present. Any ethical approvals required for the study have been obtained and are mentioned in the manuscript.

## Funding

This research did not receive any specific grant from funding agencies in the public, commercial, or not-for-profit sectors.

## Competing interests statement

All authors confirm that there are no competing interests related to financial gain, professional affiliations, intellectual property rights, or other personal considerations that could bias the findings of this research. If any potential competing interests arise, they will be disclosed to the journal immediately.

We affirm the accuracy of this declaration and acknowledge our responsibility to disclose any changes in conflict-of-interest status should they occur after submission.
